# Molecular Markers for Sensitive Detection of *Plasmodium falciparum* Asexual Stage Parasites and their Application in a Malaria Clinical Trial

**DOI:** 10.4269/ajtmh.16-0893

**Published:** 2017-04-24

**Authors:** Fitsum G. Tadesse, Kjerstin Lanke, Issa Nebie, Jodie A. Schildkraut, Bronner P. Gonçalves, Alfred B. Tiono, Robert Sauerwein, Chris Drakeley, Teun Bousema, Sanna R. Rijpma

**Affiliations:** 1 Department of Medical Microbiology, Radboud University Medical Center, Nijmegen, The Netherlands; 2 Medical Biotechnology Unit, Institute of Biotechnology, Addis Ababa University, Addis Ababa, Ethiopia; 3 Armauer Hansen Research Institute (AHRI), Addis Ababa, Ethiopia; 4 Department of Biomedical Sciences, Centre National de Recherche et de Formation sur le Paludisme, Ouagadougou,Burkina Faso; 5 Department of Immunology and Infection, London School of Hygiene and Tropical Medicine, London, United Kingdom; 6 Department of Laboratory Medicine, Radboud University Medical Center, Nijmegen, The Netherlands

## Abstract

*Plasmodium falciparum* parasite life stages respond differently to antimalarial drugs. Sensitive stage-specific molecular assays may help to examine parasite dynamics at microscopically detectable and submicroscopic parasite densities in epidemiological and clinical studies. In this study, we compared the performance of skeleton-binding protein 1 (SBP1), ring-infected erythrocyte surface antigen, Hyp8, ring-exported protein 1 (REX1), and PHISTb mRNA for detecting ring-stage trophozoite-specific transcripts using quantitative reverse transcriptase polymerase chain reaction. Markers were tested on tightly synchronized in vitro parasites and clinical trial samples alongside established markers of parasite density (18S DNA and rRNA) and gametocyte density (Pfs25 mRNA). SBP1 was the most sensitive marker but showed low-level expression in mature gametocytes. Novel markers REX1 and PHISTb showed lower sensitivity but higher specificity for ring-stage trophozoites. Using in vivo clinical trial samples from gametocyte-negative patients, we observed evidence of persisting trophozoite transcripts for at least 14 days postinitiation of treatment. It is currently not clear if these transcripts represent viable parasites that may have implications for clinical treatment outcome or transmission potential.

## Introduction

In the peripheral blood of individuals infected with *Plasmodium falciparum*, ring-stage trophozoites and mature gametocytes can be microscopically observed and their densities, directly determined, whereas mature trophozoites and schizonts are sequestered to the microvasculature of various organs^1^ and developing gametocytes accumulate in the extravascular compartment of the bone marrow.^2^ Precise quantification of circulating parasite stages is necessary to accurately assess stage-specific clearance^3^ by antimalarials and could support the evaluation of treatment efficacy^4^ in clinical trials. Indeed, artemisinin-based combination therapy (ACT), the first-line treatment against *P. falciparum* malaria, does not equally affect all malaria parasite developmental stages: although it is highly active against asexual blood stages responsible for malaria morbidity and mortality, it does not clear mature stage gametocytes, which may persist after treatment,^5^^,^^6^ often at densities below the range that is detectable by microscopy, and may allow posttreatment malaria transmission to mosquitoes.^7^^,^^8^ On the other hand, primaquine alone does not clear asexual parasites but is the only available drug that can accelerate gametocyte clearance.^9^

Several highly sensitive molecular assays for detecting *P. falciparum* parasites are currently available, including qualitative and quantitative polymerase chain reaction (PCR) targeting genomic DNA of the small subunit ribosomal RNA genes (18S) for detection and quantification of total circulating parasite biomass.^10^^,^^11^ Detection of RNA by quantitative reverse transcriptase PCR (qRT-PCR) may further increase sensitivity for highly transcribed genes and enable the specific detection of different parasite developmental stages such as gametocytes through detection of Pfs25 transcripts.^12^ This strategy can also be applied for the detection of other parasite life stages that are present in peripheral blood such as ring-stage trophozoites. An obstacle for the identification of informative trophozoite markers is the low-level expression of some candidate genes in gametocytes, which reduces stage specificity. Circulating gametocytes that persist after malaria treatment and express low-level trophozoite-specific transcripts could jeopardize molecular assays that aim to specifically detect asexual stage parasites and lead to incorrect conclusions on the effect of antimalarials on asexual parasite clearance. Ring-infected erythrocyte surface antigen (RESA) was previously used as ring-stage trophozoite marker,^13^ whereas more recently skeleton-binding protein 1 (SBP1) was proposed as highly sensitive marker for ring-stage trophozoites that is capable of detecting submicroscopic parasite densities.^14^

In the current study, we set out to compare the performances of previously reported ring-stage trophozoite markers and identify novel trophozoite-specific transcripts to establish molecular markers that can detect and quantify low densities of ring-stage trophozoites. Targets were validated using in vitro samples of synchronous early and late ring-stage trophozoites and gametocytes and field samples from a recent clinical trial where children with asymptomatic *P. falciparum* infections were treated with ACT alone or in combination with a gametocytocidal dose of primaquine.^15^

## Materials and Methods

### In vitro stage-specific parasite samples.

We obtained trophozoites at early and late ring-stage and stage V gametocytes through synchronous asexual and gametocyte culture in vitro. *Plasmodium falciparum* NF54 strain was maintained in a semiautomated culture system.^16^^–^^18^ Briefly, in vitro parasites were grown in Roswell Park Memorial Institute medium supplemented with human serum (complete medium) and 5% hematocrit. Medium was changed twice daily and fresh human red blood cells were added (Sanquin). Asexual parasites were synchronized by the selection of late ring-stage trophozoites and schizonts on a 63% percoll density gradient, which was followed by a 5% sorbitol treatment, killing the remaining schizonts after 5 hours, ensuring tight synchronization of parasites within a 5-hour window. Samples containing trophozoites at early and late ring stage were harvested 10 and 20 hours after percoll synchronization, respectively. Gametocytes were synchronized by allowing asexual cultures to grow just above 20% parasitemia, after which trophozoites and schizonts were selected on a 63% percoll density gradient. Five hours postsynchronization, 20 U/mL heparin was added to the culture medium, preventing further invasion of merozoites; thereby creating a 5-hour time window for gametocyte development and terminating asexual multiplication. Mature stage Vb gametocyte samples were obtained at day 9 postsynchronization. Parasite samples were lysed in L6 buffer^19^ and extracted immediately or frozen at −80°C until further use. L6 buffer (5.25 M GuSCN, 50 mM Tris-HCl [pH 6.4], 20 mM EDTA, 1.3% [wt/vol] Triton X-100) was prepared as described earlier.^19^

### Selection of novel ring-stage trophozoite markers and primer design and validation.

**Figure 1.**
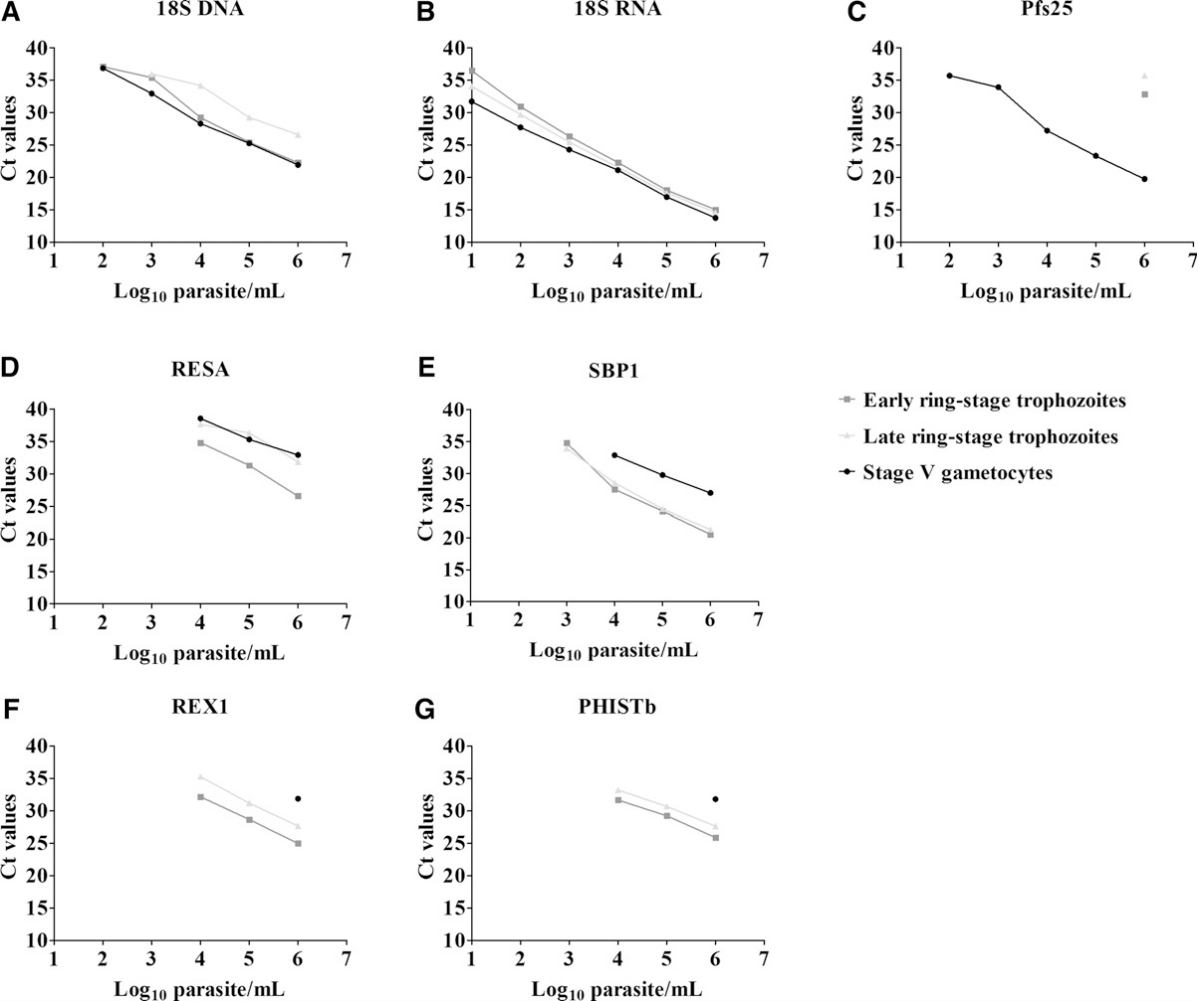
Validation of markers on stage-specific in vitro culture materials: early ring-stage trophozoites, late ring-stage trophozoites, and stage V gametocytes. Total parasitemia was measured by targeting the (**A**) 18S DNA and (**B**) 18S RNA at the transcript level. (**C**) Gametocytemic signal was quantified by targeting Pfs25. (**D**–**G**) Transcription level of early ring-stage trophozoite markers were used to quantify ring-stage trophozoites. Presented is the dilution series of duplicate measurements of the Log_10_ parasites/mL (*x* axis) and the corresponding CT values (*y* axis).

Novel ring-stage trophozoite markers were identified from a recent computational approach to determine stage specificity of *P. falciparum* transcripts,^14^ determining the ratio of early ring-stage trophozoite expression over gametocyte expression. Among the genes with the highest differential expression, we selected potential targets specific to ring-stage trophozoites that showed the highest expression levels in asexual stages, and that had an intron (Supplemental Table 1). In addition to these potential markers, we tested two previously reported ring-stage trophozoite markers, RESA (PF3D7_0102200)^20^ and SBP1^14^ by qRT-PCR. Primers were designed in an intron-spanning fashion whenever possible to avoid amplification of genomic DNA while avoiding the requirement for a digest step; multiple primer sets were tested to select primers resulting in the most sensitive, linear amplification (Supplemental Table 2 and Figure 1

). Different primers targeting Pf18S RNA and DNA, Pfs25 mRNA, SBP1 mRNA, RESA mRNA, Hyp8 mRNA, ring-exported protein 1 (REX1) mRNA, and PHISTb mRNA (Supplemental Table 3) were tested on strictly synchronized in vitro cultured asexual stage parasites (early and late ring-stage trophozoites) and stage V gametocytes.

Amplification efficiency and limit of detection of the primers were assessed by cloning the PCR-amplified products into the pTOPO-TA cloning vector (Invitrogen, Thermoscientific, Boston, MA) by strictly following the manufacturer's protocol (Supplemental Figure 1 and Table 1

**Table 1:** Correlation of markers of total parasite density or asexual parasite density with Pfs25 qRT-PCR signal

Marker	Day 0	Day 2	Day 3	Day 7	Day 10	Day 14
18S DNA	0.27 (0.001)	0.55 (0.001)	0.55 (0.001)	0.31 (0.050)	0.67 (0.001)	0.49 (0.050)
18S RNA	0.14 (0.110)	0.91 (0.001)	0.84 (0.001)	0.35 (0.010)	0.82 (0.001)	0.62 (0.001)
SBP1	0.08 (0.355)	−0.06 (0.589)	−0.01 (0.955)	−0.05 (0.820)	−0.14 (0.525)	0.01 (0.974)
REX1	0.13 (0.124)	0.17 (0.278)	−0.21 (0.380)	−0.12 (0.729)	−0.28 (0.401)	0.42 (0.201)
PHISTb	0.24 (0.010)	0.33 (0.198)	0.11 (0.629)	−0.43 (0.397)	−0.20 (0.580)	NA

NA = not available; qRT-PCR = quantitative reverse transcriptase polymerase chain reaction; REX1 = ring-exported protein 1; SBP1 = skeleton-binding protein 1. Presented is the Spearman correlation coefficient and the *P* value in parenthesis.

). Assay-specific control plasmids were run in each plate with the respective template inserted for Pf18S RNA and DNA, Pfs25, and SBP1 when the in vivo clinical trial samples were tested for the respective targets.

### Nucleic acid extraction, cDNA synthesis, and qRT-PCR methods.

Total nucleic acids were extracted from 1-mL aliquots, containing 100-μL culture material + 900-μL L6 buffer^19^ using total Nucleic Acid Isolation Kit–High Performance in a MagNAPure LC automatic extractor (Roche Applied Science, Basel, Switzerland) and eluted in 50-μL elution buffer.

For targets for which we were not able to design intron-spanning primer sets, extracted total nucleic acid material was subjected to DNA digestion with the RQ1 DNaseI (Promega, Madison, WI). cDNA was synthesized using High Capacity cDNA Reverse Transcription Kit (Applied Biosystems, Carlsbad, CA) following the manufacturers' protocols. Digestion efficiency was assessed by running a control during cDNA synthesis without the reverse transcriptase enzyme to rule out the possibility of amplification of genomic DNA, especially for targets that do not span intron regions.

All-circulating-stages parasite quantification was performed by quantitative PCR (qPCR) targeting the *P. falciparum* 18S rRNA gene on total nucleic acids, using primer and probe sequences described by Hermsen and others^21^ with minor modifications. Briefly, 5 μL of template was run in 20 μL final volume of a TaqMan Fast Advanced master Mix (Applied Biosystems) that contained 110 nM probe and 900 nM primer concentrations for 10 minutes at 95°C and for 40 cycles at 95°C for 15 seconds and 60°C for 1 minute.

qRT-PCR on SBP1, RESA, and MAL13P1.61 *Plasmodium*-exported protein (Hyp8) was run using 2.5 μL of template (cDNA) in 20 μL final volume of a GoTaq^®^ qPCR (Promega) mix that contained 225 nM primer concentration for 10 minutes at 95°C and for 40 cycles at 95°C for 15 seconds and 60°C for 1 minute and melt curve at 65°C for 5 seconds and 95°C for 50 seconds. The same protocol was followed for Pfs25 and Pf18S transcripts except that only 1 μL of template (cDNA) was run in 20 μL final volume reaction mix. For PHISTb and REX1 targets, the protocols used were similar to the Pfs25 protocol except that the annealing temperatures were 58°C and 56°C for REX1 and PHISTb, respectively.

Serial dilutions of strictly synchronized culture-derived NF54 *P. falciparum* early and late ring-stage trophozoites or stage V gametocytes and/or assay-specific control plasmids with the respective template inserted at concentrations of 10^6^–10^1^ copies of template/reaction were run in duplicate in each plate for parasite quantification. The CFX96^™^ Real-Time PCR Detection System (BIO-RAD, Hercules, CA) was used for all assays.

### Description of sample set from clinical trial.

We tested our set of molecular markers on RNA samples from a randomized, double-blind, placebo-controlled trial in which 360 children with asymptomatic malaria in seasonal and hyperendemic area of Burkina Faso were treated with artemether–lumefantrine (AL, Coartem^®^; Novartis Pharma, Basel, Switzerland), either alone or in combination with a 0.25 or 0.40 mg/kg primaquine dose.^15^ Treatment was directly observed and the trial provided evidence for faster gametocyte clearance by microscopy, Pfs25 mRNA qRT-PCR and Pfs25 mRNA quantitative nucleic acid sequence-based amplification in the primaquine arms. Finger prick blood samples (50 μL) were collected on days 0, 2, 3, 7, 10, and 14 and stored in 250 μL of RNAprotect^®^ cell reagent (QIAGEN, Hilden, Germany). Total nucleic acids were extracted as mentioned earlier using a MagNAPure LC automatic extractor and total Nucleic Acid Isolation Kit–High Performance (Roche Applied Science).

### Data analysis.

All analyses were performed with STATA 12 (StataCorp., College Station, TX) and Graph Pad Prism 5.0 (Graph Pad Software Inc., La Jolla, CA). Welch's paired Student's *t* test was performed to compare asexual stage parasite levels on different follow-up days (7–14). Levels of the different asexual stage markers and the median density of SBP1 on day 14 versus days 10 and 7 were compared using two-sample Wilcoxon rank-sum (Mann–Whitney) test. χ^2^ of Fisher's exact test was used to evaluate the prevalence of marker transcripts between the three treatment arms. Spearman's rank correlation coefficient was used to assess the relationship between asexual stage markers and the gametocyte-specific Pfs25 qRT-PCR signal.

## Results

### Selection of novel ring-stage trophozoite markers and assay performances.

In addition to the previously published ring-stage trophozoit markers, RESA (PF3D7_0102200)^13^ and SBP1,^14^ we selected targets with the highest fold change expression in asexual parasites and high ring-stage trophozoite expression levels: REX1 (PF3D7_0935900),^22^ a PHISTb-exported protein (PF3D7_0424600),^23^ and GEXP07-exported protein (Hyp8; PF3D7_1301700).^24^

Primers were developed for RESA, Hyp8, REX1, and PHISTb; published primers for 18S DNA,^21^ 18S RNA,^12^ SBP1,^14^ and Pfs25^12^ were used (Supplemental Table 3). Amplification efficiency of these primers was validated in qRT-PCRs using a dilution series of constructs containing the respective amplicon, and exceeded 91% based on slope in all cases. The limit of detection was 1–10 copies/μL for the different targets (Supplemental Table 2, not determined for REX1 and PHISTb). The cutoff value for positivity was set at a point where the PCR lost its linear detection range in more than 50% of the observations using both culture material and constructs. We were unable to obtain linear results for Hyp8 amplification despite multiple attempts to optimize conditions, disqualifying this target for ring-stage trophozoite quantification in the current study.

### Validation of specificity and sensitivity of ring-stage trophozoite and gametocyte markers using in vitro material.

The selected markers were validated on synchronous asexual parasite and gametocyte cultures providing early ring-stage trophozoites (10-hour postsynchronization), late ring-stage trophozoites (20 hours), and mature gametocyte (9 days) samples. Parasite concentrations between 10^6^ and 10^1^ parasites/mL were tested.

As expected, 18S DNA was detected in early ring-stage trophozoites, late ring-stage trophozoites, and stage V gametocytes with a limit of detection of 10 parasites/mL (Figure 1A). 18S RNA was detected with similar transcript levels in early and late ring-stage trophozoites and gametocytes (Figure 1B). Pfs25 transcripts were detected in stage V gametocytes^25^ at concentrations as low as 100 gametocytes/mL, whereas the highest ring-stage trophozoite concentration (10^6^ parasites/mL) also showed Pfs25 transcript detection. RESA transcripts were more abundantly present in ring-stage trophozoites but were also detected in stage V gametocytes with a limit of detection of ∼10,000 gametocytes/mL (Figure 1D), making it a less suitable trophozoite-specific candidate. SBP1 was detected in all parasite stages. Although transcript levels were higher in ring-stage trophozoites, gametocyte concentrations ≥ 10,000 gametocytes/mL had detectable SBP1 transcripts in our in vitro assays (Figure 1E). REX1 was specific for ring-stage trophozoites; only the highest concentration of gametocytes (10^6^ parasites/mL) showed a detectable signal for REX1 (Figure 1F). However, REX1 was generally less sensitive for ring-stage trophozoite detection compared with other markers with a limit of detection of 10,000 parasites/mL (Figure 1F). PHISTb shared a similar specificity with REX1, detecting exclusively asexual parasites (Figure 1G) and only parasite densities ≥ 10,000 parasites/mL had detectable amplification.

### Stage composition of parasites after artemether–lumefantrine treatment with or without primaquine.

**Figure 2.**
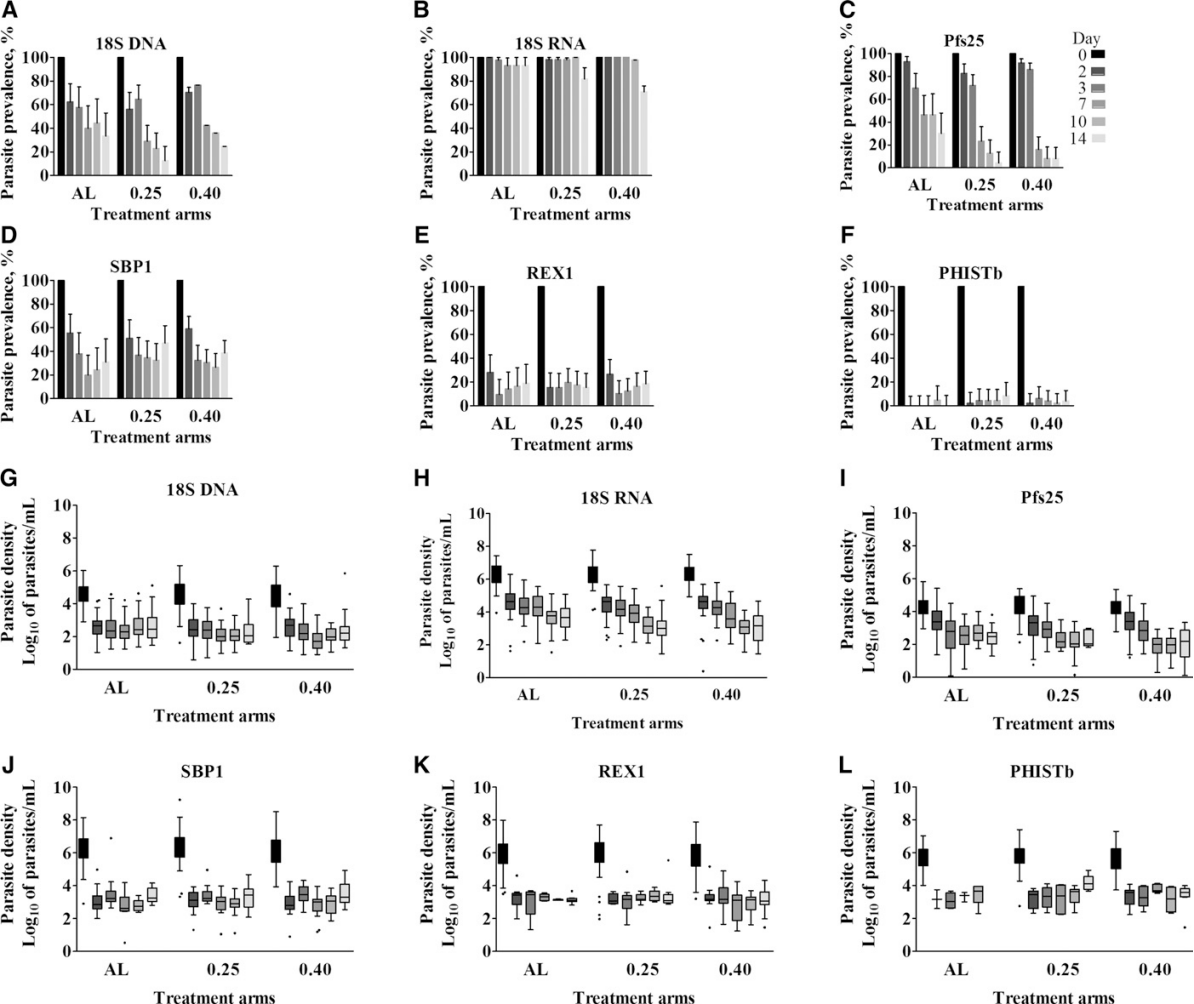
Parasite carriage and stage composition of clinical malaria trial samples. Prevalence of (**A**–**B**) total parasites, (C) gametocytes, and (**D**–**F**) asexual parasites in the three treatment arms is presented. Bar graphs indicate relative prevalence of the targets with regard to the baseline signal on day 0 vs. days 2, 3, 7, 10, and 14 after initiation of treatment. (**G**–**L**) Box plots in the lower panel indicate the median, 25th and 75th percentile parasite densities (Log_10_ parasites/mL) of the markers in the three treatments arms. Whiskers indicate the 5th and 95th percentiles. The three treatment groups are presented on the *x* axis: AL = only artemether–lumefantrine, 0.25 = artemether–lumefantrine + 0.25 mg/kg primaquine, and 0.40 = Arthermer Lumefantrine + 0.40 mg/kg primaquine. Shading of bars and whiskers indicates days since the start of treatment (days 0–14), dark to light colors. Error bars indicate the upper limit of the 95% confidence interval of a binomial distribution.

The complete set of molecular markers comprising 18S DNA, 18S RNA, Pfs25, SBP1, REX1, and PHISTb was used to evaluate parasite carriage and stage composition in samples from a clinical malaria trial.^15^ Prevalence of parasite carriage was determined as the percentage of samples for which a signal was detected with CT value below the assay-specific cutoff, and density in positive samples was estimated using in vitro standards. In Figure 2A and B

, prevalence of parasites was determined using qPCR and qRT-PCR to detect genomic 18S DNA or 18S transcripts, respectively. Parasite density according to 18S DNA or 18S RNA signal is depicted in Figure 2G and H. Both prevalence and density of parasites determined by 18S qPCR decreased rapidly after treatment although parasites were detected in a proportion of study participants until day 14 (Figure 2A and G). In the groups receiving primaquine, 18S qPCR parasite prevalence was lower at day 7 and beyond (*P* < 0.01, Table 2

**Table 2:** Parasite prevalence by different molecular markers after initiation of treatment with artemether–lumefantrine alone or in combination with primaquine

Markers	Days since treatment	AL	AL + 0.25 mg/kg PQ	AL + 0.40 mg/kg PQ	AL vs. AL + 0.25 mg/kg PQ			AL vs. AL + 0.40 mg/kg PQ			*P* values overall
		% (*n*/*N*)	% (*n*/*N*)	% (*n*/*N*)	*P* values	OR	95% CI	*P* values	OR	95% CI	
Parasite prevalence by 18S DNA											
	3	60.5 (26/43)	63.3 (31/49)	50.0 (26/52)	0.783	1.13	0.48, 2.62	0.309	0.66	0.29, 1.48	0.3563
	7	42.9 (18/42)	28.0 (14/50)	9.26 (5/54)	0.138	0.52	0.22, 1.23	0.001	0.14	0.05, 0.41	0.001
	10	48.8 (20/41)	22.0 (11/50)	18.9 (10/53)	0.009	0.3	0.12, 0.73	0.003	0.24	0.10, 0.61	0.0037
	14	36.6 (15/41)	12.2 (6/49)	11.1 (6/54)	0.009	0.24	0.08, 0.70	0.005	0.22	0.08, 0.63	0.004
Parasite prevalence by 18S RNA											
	3	97.7 (42/43)	98.0 (48/49)	98.1 (51/52)	0.926	1.14	0.07, 18.84	0.892	1.21	0.07, 20.00	0.9905
	7	95.2 (40/42)	96.0 (48/50)	92.6 (50/54)	0.859	1.2	0.16, 8.90	0.598	0.62	0.11, 3.59	0.731
	10	97.6 (40/41)	98.0 (49/50)	88.7 (47/53)	0.887	1.23	0.07, 20.21	0.139	0.2	0.02, 1.70	0.0761
	14	97.6 (40/41)	81.6 (40/49)	63.0 (34/54)	0.041	0.11	0.01, 0.92	0.003	0.04	0.01, 0.33	0.001
Gametocyte prevalence by Pfs25											
	3	69.8 (30/43)	69.4 (34/49)	82.7 (43/52)	0.969	0.98	0.40, 2.39	0.141	2.07	0.79, 5.46	0.2091
	7	47.6 (20/42)	22.0 (11/50)	14.8 (8/54)	0.011	0.31	0.13, 0.76	0.001	0.19	0.07, 0.50	0.0013
	10	48.8 (20/41)	12.0 (6/50)	7.6 (4/53)	0.001	0.14	0.05, 0.41	0.001	0.09	0.03, 0.28	0.001
	14	31.7 (13/41)	4.1 (2/49)	7.4 (4/54)	0.003	0.09	0.02, 0.44	0.004	0.17	0.05, 0.58	0.0004
Asexual parasite prevalence by SBP1											
	3	39.5 (17/43)	36.7 (18/49)	30.8 (16/52)	0.783	0.89	0.38, 2.06	0.373	0.68	0.29, 1.59	0.6529
	7	21.4 (9/42)	34.0 (17/50)	27.8 (15/54)	0.185	1.89	0.74, 4.84	0.477	1.41	0.55, 3.64	0.4047
	10	26.8 (11/41)	32.0 (16/50)	24.5 (13/53)	0.591	1.28	0.52, 3.19	0.8	0.89	0.35, 2.25	0.692
	14	34.2 (14/41)	46.9 (23/49)	35.2 (19/54)	0.22	1.71	0.73, 4.01	0.92	1.05	0.45, 2.46	0.36
Asexual parasite prevalence by REX1											
	3	9.3 (4/43)	14.3 (7/49)	9.6 (5/52)	0.465	1.63	0.44, 6.00	0.959	1.04	0.26, 4.13	0.6921
	7	14.3 (6/42)	18.0 (9/50)	11.1 (6/54)	0.632	1.32	0.43, 4.06	0.642	0.75	0.22, 2.52	0.606
	10	17.1 (7/41)	16.0 (8/50)	15.1 (8/53)	0.891	0.93	0.30, 2.81	0.795	0.86	0.29, 2.61	0.9669
	14	19.5 (8/41)	14.3 (7/49)	16.7 (9/54)	0.51	0.69	0.23, 2.1	0.72	0.83	0.29, 2.36	0.8
Asexual parasite prevalence by PhISTb											
	3	0 (0/43)	4.1 (2/49)	5.8 (3/52)	NA	NA	NA	0.697	0.7	0.11, 4.35	0.6948
	7	0 (0/42)	4.0 (2/50)	3.7 (2/54)	NA	NA	NA	NA	NA	NA	0.9374
	10	4.9 (2/41)	4.0 (2/52)	1.9 (1/53)	0.839	0.81	0.11, 6.03	0.43	0.38	0.03, 4.29	0.6943
	14	0 (0/41)	8.2 (4/49)	3.7 (2/54)	0.35	2.31	0.40, 13.21	NA	NA	NA	0.33

AL = artemether/lumefantrine; CI = confidence interval; NA = not available; OR = odds ratio; PQ = primaquine.

).^15^ The 18S RNA transcripts suggest a much higher prevalence of parasite-positive individuals after treatment and decrease only on 14 days after initiation of treatment with lower prevalence in the primaquine arms (*P* < 0.001, Table 2). Gametocyte prevalence, determined by the number of individuals positive for Pfs25 transcripts, decreased following treatment in all arms with lower day 7 gametocyte prevalence in the primaquine-treated groups (*P* < 0.001, Table 2). Gametocyte density in gametocyte-positive samples did not decrease in a dose-dependent manner (Figure 2I).

The expression of asexual stage-specific markers SBP1, REX1, and PHISTb (Figure 2D–F) similarly indicate that a fraction of the study population remains parasite positive until the end of the study at day 14. The prevalence at days 7, 10, and 14 is higher for SBP1 (20–40%) compared with REX1 (10–20%) and PHISTb (0–5%) (Table 2). This fraction does not decrease in a time-dependent manner, and is similar between study arms (Table 2). There was no statistically significant difference in mean asexual parasite density between different time points for all markers except SBP1 (Figure 2J–L). The mean density of asexual parasites, quantified by SBP1 qRT-PCR appeared to increase toward the end of follow-up (Table 3

**Table 3:** Asexual parasite densities estimated by SBP1 on days 7, 10, and 14 after initiation of treatment

Treatment arm	Days since treatment	*N*	Median (25th–75th percentiles)	*P* value
Combined	14	53	1,741.8 (805.4–6025.6)	
	10	41	668.3 (309.7–1710.0)	0.0062
	7	43	1,074 (323.4–2,275.1)	0.0162
AL	14	10	1,706.1 (1,081.4–2,296.1)	
	10	5	447.7 (291.7–937.6)	0.1171
	7	8	817 (308.1–2,895.1)	0.778
AL + 0.25 mg PQ	14	22	2,230.8 (409.3–3,033.9)	
	10	20	670 (439.6–1,565.1)	0.1341
	7	17	1,074 (339.6–1,798.9)	0.2345
AL + 0.40 mg PQ	14	21	1,990.7 (977.2–10,568.2)	
	10	16	887.5 (220.6–2,338.9)	0.0385
	7	18	1,087.9 (282.5–1,584.9)	0.0408

AL = artemether/lumefantrine; *N* = number of samples positive for SBP1; PQ = primaquine; SBP1 = skeleton-binding protein 1. Density is expressed as estimated number of parasites per milliliter of blood. The *P* value reflects the difference in density between day 14 and days 7 and 10.

) and was higher on day 14 compared with days 7 and 10 (*P* < 0.001 and *P* < 0.01, respectively). Parasite density determined by PHISTb expression, however, was significantly higher compared with SBP1-determined densities (*P* = 0.03) (Figure 2L). This may be due to the lower sensitivity of PHISTb, resulting in quantitative data for higher parasite density samples only.

### Persisting ring-stage trophozoite transcripts in relation to posttreatment gametocyte carriage.

**Figure 3.**
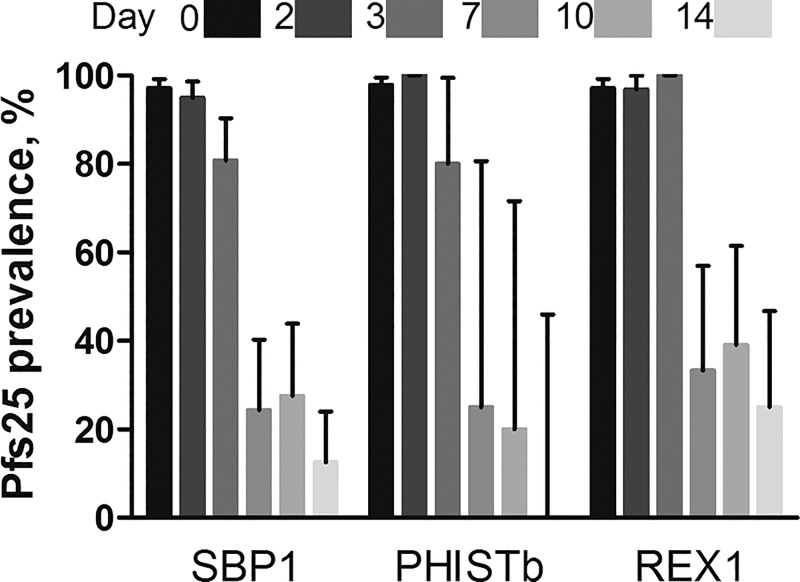
Pfs25 prevalence in samples obtained at the different time points that were positive for skeleton-binding protein 1 (SBP1), ring-exported protein 1 REX1, or PHISTb. Bar graphs indicate the relative gametocytes signal on day 0 vs. days 2, 3, 7, 10, and 14 after initiation of treatment among samples that were found positive with the three targets. Shading of bars indicates days since the start of treatment (days 0–14), dark to light colors. Error bars indicate the upper limit of the 95% confidence interval of a binomial distribution.

**Figure 4.**
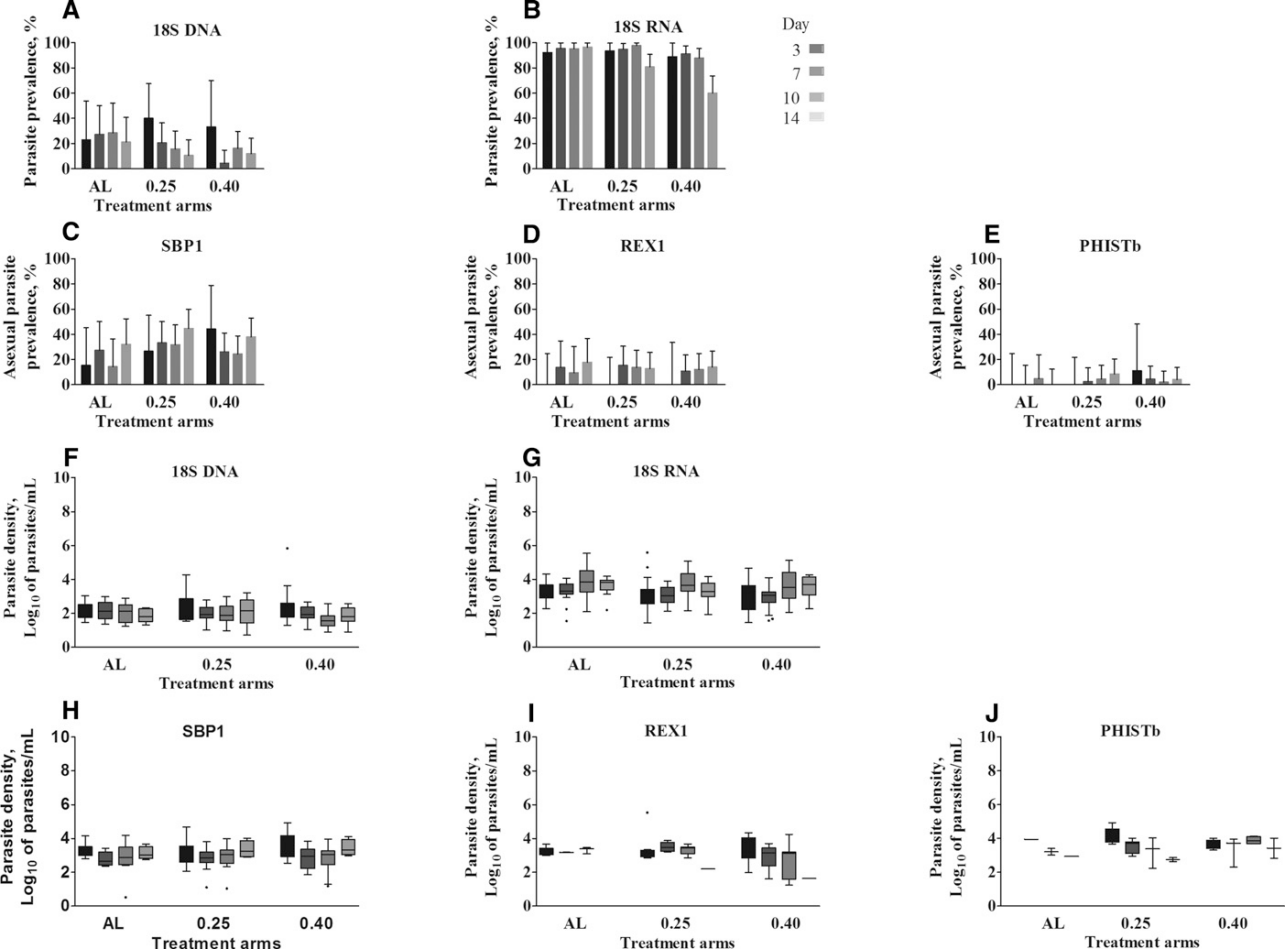
Persisting parasite signal in the absence of gametocytemia in clinical trial samples. Prevalence of (**A**–**B**) total parasites and (**C**–**E**) ring-stage trophozoites of clinical malaria trial samples that was negative for gametocyte transcripts (Pfs25). Presented in the bar graphs is the prevalence of the signal with the different markers in different days since treatment, in the three treatment arms. (**F**–**J**) Box plots indicate the median, 25th and 75th percentile parasite densities (Log_10_ parasites/mL) of the markers in the three treatments arms. Whiskers indicate the 5th and 95th percentiles. The three treatment groups are presented on the *x* axis: AL = only artemether–lumefantrine, 0.25 = AL + 0.25 mg/kg primaquine, and 0.40 = AL + 0.40 mg/kg primaquine. Shading of bars and whiskers indicates days since the start of treatment (days 3–14), dark to light colors. Error bars indicate the upper limit of the 95% confidence interval of a binomial distribution.

In addition to our in vitro assessments of ring-stage trophozoite transcripts in mature gametocytes, we quantified the correlation between gametocyte markers and our general parasite markers and ring-stage trophozoite markers to explore the possible contribution of persisting gametocytes to ring-stage trophozoite transcripts. As anticipated, the gametocyte signal correlated with the total parasite signal based on 18S DNA and transcripts on all days, except at day 0 (Table 1). The signal of ring-stage trophozoite markers was not correlated with the gametocyte signal during the entire period of follow-up, confirming that they detect different parasite populations. To further exclude the possibility of background expression by gametocytes contributing to the asexual marker signal, we determined Pfs25 prevalence in samples obtained at the different time points that were positive for SBP1, REX1, or PHISTb (Figure 3

). With the exception of enrolment and day 3 postinitiation of treatment, when the majority of individuals were gametocyte positive,^26^ a minority of SBP1-, REX1-, and PHISTb-positive samples concurrently had gametocytes detected by Pfs25 qRT-PCR. As REX1 and PHISTb are highly specific for asexual parasites with detection of gametocytes expression only at concentrations exceeding 10^5^ parasites/mL, this suggests that there is no relevant contribution of gametocytes to trophozoite-specific signals posttreatment. In addition, in samples where Pfs25 signal could not be detected, we still detected total parasites with Pf18S DNA and RNA and ring-stage trophozoite signals with the three markers (SBP1, REX1, and PHISTb) (Figure 4A–E

). Overall, 34.5% (132/383) of samples that were negative for gametocytes from day 3 after treatment onward were positive for one or more of the erring-stage trophozoite markers (Table 4

**Table 4:** Agreement between SBP1, REX1, and PHISTb for detecting asexual ring-stage parasite prevalence

		SBP1		PHISTb	
		Positive	Negative	Positive	Negative
REX1	Positive	10.1 (35/346)	3.2 (11/346)	1.7 (6/346)	11.6 (40/346)
	Negative	21.4 (74/346)	65.3 (226/346)	2.0 (7/346)	84.7 (293/346)
PhISTb	Positive	3.2 (11/346)	0.6 (2/346)		
	Negative	28.3 (98/346)	67.9 (235/346)		

REX1 = ring-exported protein 1; SBP1 = skeleton-binding protein 1. Observations from days 7, 10, and 14 are combined to give one denominator. Presented is the percentage of samples that is positive for a given marker (*n*/*N*).

). Corresponding parasite densities for samples without gametocyte-specific Pfs25 signal at days 3, 7, 10, and 14 are depicted in Figure 4H–J, respectively.

### Specificity of SBP1 for asexual parasite detection.

The agreement between ring-stage trophozoite detection by SBP1, REX1, and PHISTb was determined in follow-up day 7–14 samples that were gametocyte negative (Table 4). SBP1 was able to identify more individuals, 31.5% (109/346), with trophozoite-specific transcripts compared with REX1 and PHISTb, 13.3% (46/346) and 3.8% (13/346), respectively. SBP1 qRT-PCR was positive for 89.3% (109/122) of all samples with evidence of ring-stage trophozoites by any of the three assays, the remaining samples being detected only by REX1 (9.0%; 11/122) or PHISb (1.6%; 2/122). We observed moderate but highly significant agreement between positivity by SBP1 and REX1 (agreement = 75.4%, kappa = 0.33; *P* < 0.0001) and PHISTb and REX1 (agreement = 86.4%, kappa = 0.15; *P* < 0.001) and lower agreement between SBP1 and PHISTb (agreement = 71.1%, kappa = 0.12; *P* < 0.0001) (Table 4).

## Discussion

Herein, we examined the sensitivity and stage specificity of five markers (RESA, Hyp8, SBP1, REX1, and PHISTb) for ring-stage trophozoites alongside several markers of total parasitemia and gametocytemia. Our assays highlight the complexity of interpreting RNA transcript data in clinical trial samples. Our combination of in vitro time series and an in vivo comparison of markers for the evaluation of an antimalarial drug trial indicates that in a proportion of patients, ring-stage trophozoite transcripts persist for 14 days after treatment with ACTs plus different doses of primaquine.

ACTs rapidly reduce the asexual parasite biomass that is present on clinical presentation with *P. falciparum* malaria. Nevertheless, clinical trials have reported molecular signals indicative of persistence of asexual stage parasites for several weeks.^4^^,^^27^^–^^29^ It is currently unclear whether these are true indications of persisting asexual parasites and, until now, artemisinin resistance has not been demonstrated in Africa.^30^ Before the current study, the stage specificity of these markers had not been rigorously examined with, for example, conflicting evidence on the stage specificity of SBP1.^4^ At present, there is no consensus on the value of molecular tools to detect asexual parasites with high sensitivity and specificity or interpretation of RNA transcripts after antimalarial treatment.^4^ The sensitivity of pathogen detection by molecular assays depends on the blood volume examined, the target, and assay efficiency.^31^ Although RNA targets may be unstable and particularly prone to contamination challenges,^32^ the detection of abundant RNA transcripts allows highly sensitive stage-specific detection of parasites.

Our in vitro experiments confirm that DNA targets and RNA transcripts of the 18S RNA gene are abundantly present in both asexual parasites and gametocytes.^25^ Posttreatment persistence of 18S DNA or RNA in peripheral blood can thus be a consequence of the presence of both trophozoites and gametocytes. The faster decline in the prevalence of both Pf18S DNA and RNA in the groups that received primaquine treatment is thus likely to be due to a faster clearance of gametocytes following primaquine.^15^^,^^33^ This is supported by a decrease in Pfs25-based gametocyte prevalence, which was significantly lower in the primaquine-treated groups at days 7, 10, and 14 postinitiation of treatment.^15^ We found detectable signals of the mature gametocytes stage-specific marker, Pfs25, in both early and late ring-stage trophozoites when these asexual parasites were present at densities ≥ 10^6^ parasites/mL in vitro. Very low levels of Pfs25 mRNA were previously reported in synchronous asexual parasite culture.^28^ Our findings suggest that high asexual parasitemia may be accompanied by a false positive Pfs25 gametocyte signal. This may lead to overestimations of baseline gametocyte prevalence in clinical malaria cases that typically have higher parasite densities but is unlikely to affect estimates of posttreatment gametocyte dynamics when the asexual parasite biomass is typically reduced by > 99% within days after initiation of treatment.^34^

Our work confirms that gametocytes commonly persist in low concentrations for several weeks after treatment with ACT or ACT with primaquine.^6^^,^^35^ This illustrates the necessity to develop molecular assays that specifically detect asexual parasites against a background of persisting gametocytes that are epidemiologically relevant but not associated with malaria pathogenesis or treatment failure. Understanding and minimizing the impact of gametocytemia on asexual parasite detection is essential to allow molecular assays to support the interpretation of drug efficacy in clinical trials. We thus aimed to identify gene targets with negligible transcript levels in gametocytes but sensitive detection of ring-stage trophozoites. Our selection of markers included two previously reported^13^^,^^14^ and newly identified markers. SBP1, REX1, and PHISTb were considered the most promising candidates based on their performance in tightly synchronized in vitro culture material. Our criteria included detectability of asexual parasites at densities below 1,000 parasites/mL, the approximate detection threshold for nested PCR or qPCR.^31^ Although the exact lower limit of detection is difficult to extrapolate from in vitro studies to field settings, our approach allowed comparison of target genes and primer sequences for these genes. Among the three candidates that were promising in in vitro experiments, SBP1 was the most sensitive^4^^,^^14^ but also showed the most pronounced expression in stage V gametocytes. The other two newly identified markers, REX1 and PHISTb, showed high specificity for ring-stage trophozoites; as only gametocyte densities ≥ 10^6^ gametocytes/mL were detectable. Gametocyte densities in symptomatic and asymptomatic infections are typically below 10^4^ gametocytes/mL.^36^^,^^37^ If transcript levels are comparable between in vitro-cultured parasites and natural infections, it is thus unlikely that a positive signal in REX1 and PHISTb qRT-PCR assays is caused by gametocytes in natural infections, with a possible exception for rare high-density gametocyte carriers. However, this increased specificity of REX1 and PHISTb compared with SBP1 is challenged by a lower sensitivity: SBP1 was able to detect lower concentrations of asexual parasites compared with REX1 and PHISTb based in in vitro experiments.

SBP1, REX1, and PHISTb were used to investigate the presence of persisting asexual parasites in a clinical trial with high treatment efficacy and no asexual parasites detected by microscopy beyond day 2 after initiation of treatment.^15^ All three markers suggested that low densities of asexual parasites persisted after treatment. We chose a highly conservative approach to minimize the chances of a contribution of gametocytes to this finding. After excluding all Pfs25-positive samples, and thereby all samples with > 0.1 gametocytes/μL, 43.2% (*n*/*N*) of samples remained positive for the different markers on day 14 postinitiation of treatment. SBP1 was the most sensitive of our tested markers for detecting ring-stage trophozoites with low to moderate agreement with PHISTb and REX1 assays. An important caveat is that our markers were selected based on expression in a single laboratory-adapted parasite line, NF54. Although we previously assessed SBP1 transcript levels in 3D7^4^ and Pfs25 transcript levels in NF54, NF135, and NF166 parasites^38^ and found highly comparable levels between parasite isolates, this does not mean that in vitro expression can be directly extrapolated to expression in in vivo samples. Our quantitative approach may thus be affected by possible differences between in vitro-cultured parasites and patient samples. In future studies that use transcripts for parasite quantification, comparing expression profiles of target genes between parasite strains and from parasites extracted in vivo can address this uncertainty.

It is currently unknown whether our ring-stage trophozoite transcripts represent viable malaria parasites and whether mRNA transcripts may linger in the circulation after asexual parasites have successfully been cleared. Injection of killed *Plasmodium chabaudi* parasites directly into the bloodstream or parasite remnants derived from parasites cleared under natural conditions do not contribute to a signal that is investigated with DNA-based PCR,^39^ implicating that DNA derived from dead parasites circulates for less than 48 hours. This same study, however, reported that live but drug-damaged parasites (which would be unable to initiate an infection when re-inoculated) may contribute to a positive PCR signal in mice that were infected with *P. chabaudi* and treated with antimalarial drug, up until days 13–16. On the other hand, in other diseases such as Leishmania it was demonstrated that DNA is rapidly degraded following parasite death so that PCR detection indicates viable cells.^40^

In malaria, parasites are removed by circulating and reticuloendothelial phagocytes,^41^ removing parasite nuclear material from the circulation. ACTs activate the host system, mainly spleen, for a process called “pitting” to facilitate the rapid clearance of asexual parasites.^42^^,^^43^ Taken together, these observations suggest that nonviable cells are unlikely to give RT-PCR–positive signals. Arguing against the suggestion of viable parasites being detected by our asexual markers is the finding in one Ugandan study that SBP1 transcripts were not predictive of treatment failure during 28 days of follow-up^4^ or microscopy detectable rises in parasite density.^33^ We did, however, find indications that parasite density as quantified by SBP1 qRT-PCR increased in individuals on day 14, a moment that protective plasma concentrations of lumefantrine wane.^44^ This finding has to be interpreted with caution since no treatment failure was observed. Longer periods of follow-up may be needed to examine this. In addition, ex vivo parasite culture from posttreatment blood samples may provide evidence on the viability of the low-density parasite populations whose presence our RNA-based assays suggests.

We aimed at elucidating stage-specific parasite dynamics after antimalarial treatment using an array of quantitative molecular assays. The high abundance and persistence of 18S RNA transcripts renders this target uninformative to interpret treatment efficacy. Our findings also demonstrate the complexity of interpreting qRT-PCR findings since none of the examined targets was exclusively transcribed in asexual parasites or gametocytes. Three targets that show high specificity for ring-stage trophozoites, and have only low levels of transcripts in mature gametocytes, all indicate persisting transcripts until day 14 postinitiation of treatment. Our duration of follow-up was chosen to inform the gametocytocidal properties of the different antimalarials^15^ and was too short to determine whether the persistence of trophozoite-specific transcripts was associated with subsequent recrudescence of infections,^45^ de novo production of gametocytes or enhanced transmission potential.^27^ Our study has demonstrated the strength and weaknesses of several asexual parasite targets for epidemiological and clinical studies. Future studies should confirm their sensitivity in natural infections and determine the relevance of persisting parasite transcripts after antimalarial treatment.

## Supplementary Material

Supplemental Table and Figures.
