# Supplementary material for: Molecular Markers for Sensitive Detection of Plasmodium falciparum Asexual Stage Parasites and their Application in a Malaria Clinical Trial
Source: Am J Trop Med Hyg. 2017 Apr 24;97(1):188–98. doi: 10.4269/ajtmh.16-0893 (PMC5508903; doi:10.4269/ajtmh.16-0893)
Supplement: Supplementary file 1 [file tpmd160893.SD1.pdf]

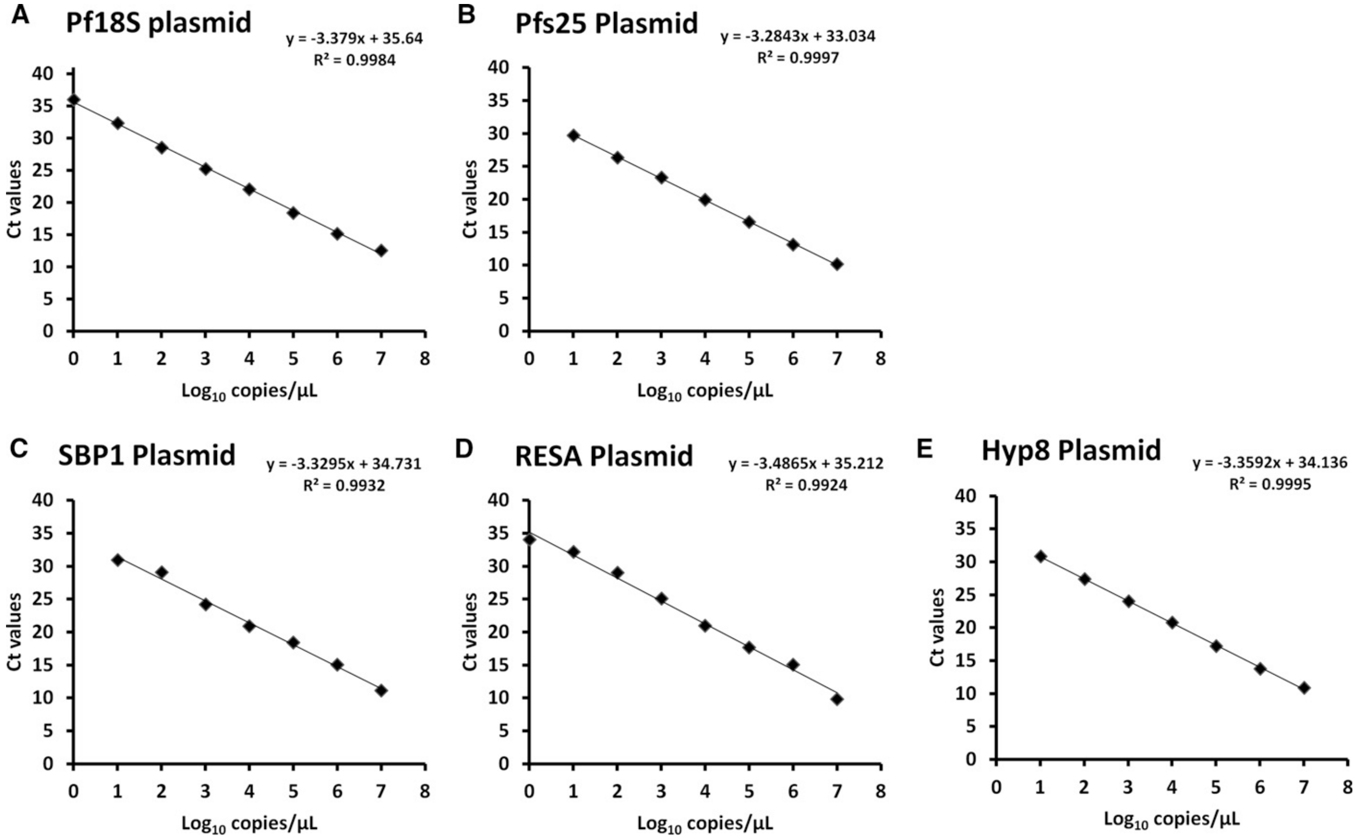

Supplemental Figure 1. Amplification curves. The linear regression for the amplification curves of (**A**) Pf18S DNA, (**B**) Pfs25, (**C**) SBP1, (**D**) RESA, and (**E**) Hyp8 to allow an assessment of polymerase chain reaction efficiency. Presented is the CT values (*y* axis) and the corresponding Log_{10} transformed plasmid copy numbers per µL (*x* axis). Amplification efficiency was determined from the slope of the linear curve (indicated on the top right corners).

Supplemental Table 1

Selection of candidate marker

GeneID	Frequency	Intron	Beta-ring stages	Beta-mature gametocytes	Product description	Rings/MG
PFI1735c	6	1	11.04258	2.152472	Ring-exported protein 1 (REX1)	5.130184
PF10_0016	5	0	5.538458	1.0868	Acyl-CoA binding protein, isoform 2, ACBP2	5.096117
PFD1170c	24	1	8.970933	2.077469	*Plasmodium*-exported protein (PHISTb), unknown function	4.318204
PFL2560c	2	1	8.053292	1.893178	*Plasmodium*-exported protein, unknown function	4.253848
MAL13P1.273	2	0	3.206335	0.784526		4.086971
PF08_0003	8	1	5.818946	1.461252	Tryptophan/threonine-rich antigen	3.982164
PFD0095c	0	1	6.810232	1.906783	*Plasmodium*-exported protein (PHISTb), unknown function	3.571582
MAL7P1.6	1	1	9.048624	2.575236	*Plasmodium*-exported protein (hyp12), unknown function	3.513707
MAL13P1.61	17	1	11.47698	3.29632	*Plasmodium*-exported protein (hyp8), unknown function	3.481757
MAL7P1.171	16	1	8.620577	2.562053	*Plasmodium*-exported protein, unknown function	3.364715
PFB0095c	18	1	9.215172	2.805933	Erythrocyte membrane protein 3	3.284175
PF10_0022	18	1	5.936012	1.838252	*Plasmodium*-exported protein (PHISTc), unknown function	3.229161
PFB0256w	12	0	3.34374	1.042435		3.207625
PFC0055w	11	1	2.23945	0.700442	*Plasmodium*-exported protein (hyp13), unknown function	3.197195
PF11_0503	13	1	9.200204	2.914172	*Plasmodium*-exported protein (PHISTc), unknown function	3.157056
MAL7P1.170	1	1	11.19367	3.556579	*Plasmodium*-exported protein, unknown function	3.147315

Supplemental Table 2

Primer efficiency and limit of detection

Target	Slope	Efficiency (%)	LOD (copies/μL)	R^2	Cutoff CT values for positivity
Pf18S DNA	–3.3790	98	1	0.9984	36.0
Pfs25	–3.2845	102	10	0.9997	33.0
SBP1	–3.3295	100	10	0.9932	34.5
RESA	–3.4865	94	1	0.9924	ND
Hyp8	–3.3592	91	1	0.9995	ND
PHISTb	ND	ND	ND	ND	35.0
REX1	ND	ND	ND	ND	35.0

LOD = limit of detection; ND = not determined; RESA = ring-infected erythrocyte surface antigen; REX1 = ring-exported protein 1; SBP1 = skeleton-binding protein 1. Primer efficiency and limit of detection were assessed by cloning polymerase chain reaction products of the respective primers into p-TOPO-TA vectors. Constructs were used as quantitative controls/1standards as well.

Supplemental Table 3

Primer sequence

Primer	GeneID	Direction	Sequence	Annealing temperature (°C)	Reference
18S DNA	PF3D7_1148600	Forward	GTAATTGGAATGATAGGAATTTACAAGGT	60	21
		Reverse	TCAACTACGAACGTTTTAACTGCAAC		
18S RNA	PF3D7_1148600	Forward	TCCGATAACGAACGAGATCTTAAC	60	12
		Reverse	ATGTATAGTTACCTATGTTCAATTTCA		
Pfs25	PF3D7_1031000	Forward	GAAATCCCGTTTCATACGCTTG	60	12
		Reverse	AGTTTTAACAGGATTGCTTGTATCTAA		
SBP1	PF3D7_0501300	Forward	GCAAAACAAGCCGTACATGTTG	60	14
		Reverse	TTGCTAGGTAATATCCTTTTCTTTTTCC		
RESA	PF3D7_0102200	Forward	CAGCATATGGGTTTACTGGC	60	
		Reverse	TGGAAGTTCATCTTCTGGCGTACA		
REX1	PF3D7_0935900	Forward	GCTCCCTTTATTCTTGTG	58	
		Reverse	CATTATCATTTCCCTTCC		
PHISTb	PF3D7_0424600	Forward	GGATAGCAAGAATCTTAAGAAC	56	
		Reverse	AACATACGTTCAATAAGACAAC		
Hyp8	Pf3D7_1301700	Forward	CTTAATAATGGAAACTTTAAGAAC	60	
		Reverse	GTGTAGATTGTTCTGTTTCGAC		

RESA = ring-infected erythrocyte surface antigen; REX1 = ring-exported protein 1; SBP1 = skeleton-binding protein 1.
